# Highly Sensitive and Specific Lateral Flow Detection for DNA Methylation Based on GIaI-Mediated Specific-Terminal-Mediated Polymerase Chain Reaction

**DOI:** 10.3390/mi16040387

**Published:** 2025-03-28

**Authors:** Lihui Ke, Hang Zhao, Hongbo Shan, Yicheng Chen, Yongsheng Cai, Yang Wang, Bo Wei, Minghua Du

**Affiliations:** 1 Department of Thoracic Surgery, Beijing Tiantan Hospital, Capital Medical University, Beijing 100070, China; k_ever96@163.com (L.K.); cysxdys@163.com (Y.C.); 2 College of Health Science and Environmental Engineering, Shenzhen Technology University, Shenzhen 518118, China; zhaohang@sztu.edu.cn; 3 Adicon Clinical Laboratories, Inc., Hangzhou 310023, China; aillen.shan@adicon.com.cn; 4 Key Laboratory of Biomechanics and Mechanobiology, Ministry of Education, Beijing Advanced Innovation Center for Biomedical Engineering, School of Engineering Medicine, Beihang University, Beijing 100191, China; ycchen@buaa.edu.cn; 5 Department of Emergency, The First Medical Center, Chinese PLA General Hospital, Beijing 100853, China

**Keywords:** DNA methylation detection, STEM-PCR, methylation-dependent restriction endonucleases, *Septin 9*

## Abstract

Sensitive and specific detection of DNA methylation is crucial for the early diagnosis of various human diseases, particularly cancers. However, conventional methylation detection methods often face challenges in balancing both sensitivity and specificity. In this study, we present a novel approach that integrates the high specificity of methylation-dependent restriction endonuclease (GlaI) digestion with the amplification efficiency of specific terminal-mediated polymerase chain reaction (STEM-PCR). This combination enables selective amplification of methylated DNA, which is then detected through lateral flow detection (LFD), providing a simple, visual readout. As a proof of concept, a STEM-PCR-LFD assay was applied to detect methylated *Septin 9*, a biomarker for colorectal cancer. The assay demonstrated a sensitivity of approximately 0.1% (10 copies of methylated template per reaction), with no cross-reactivity observed when 10,000 copies of unmethylated DNA were included as background. Furthermore, the assay was validated with ten formalin-fixed paraffin-embedded (FFPE) tissue samples, achieving 100% consistency with standard real-time STEM-PCR. This method offers a highly sensitive, specific, and accessible platform for DNA methylation detection, with potential for early disease diagnosis.

## 1. Introduction

DNA methylation is a prevalent epigenetic modification in which cytosine is converted into 5-methylcytosine (5-mC) by the action of DNA methyltransferases [1,2,3]. Among these, DNA methylation on CpG islands has been extensively studied and is known to play critical roles in gene transcription regulation and chromosomal inactivation, processes that are integral to disease development, including cancer [4,5]. Aberrant CpG methylation, particularly within gene regulatory regions, has been identified as a key driver of tumor progression [6]. Consequently, alterations in DNA methylation patterns are regarded as promising biomarkers for cancer diagnosis and prognosis [7,8]. However, detecting tumor-associated methylated DNA is challenging due to its low abundance relative to unmethylated DNA [9], necessitating highly sensitive and specific detection methods for clinical and biomedical applications [10].

Currently, bisulfite (BS) conversion remains the gold standard for DNA methylation detection [11,12,13]. This method converts unmethylated cytosines into uracils while leaving methylated cytosines unaltered, enabling downstream detection techniques, such as methylation-specific PCR (MSP) [14], isothermal amplification [15], Methylight [16], and gene sequencing [17,18]. Despite its widespread use, BS conversion-based assays suffer from several limitations, including labor-intensive workflows, DNA degradation that compromises sensitivity, and incomplete conversion, leading to false-negative results [19,20,21,22].

Methylation-dependent restriction endonucleases (MDREs) provide an alternative BS-free approach with excellent specificity and milder reaction conditions [23]. These enzymes selectively recognize and digest methylated DNA, leaving unmethylated DNA intact [24]. Among MDREs, GlaI has emerged as a novel enzyme with ultrahigh specificity for methylated sites, which is capable of recognizing a specific DNA sequence (5′-G/GATCC-3′) and cleaving it. The cleavage site is located between G and GATCC, resulting in sticky ends [25,26]. Given the ultra-low abundance of methylated sequences in the DNA pool, DNA amplification strategies are often employed to enhance the sensitivity of detecting GlaI-digested templates [27]. Methods such as isothermal exponential amplification reaction (EXPAR) [27] and helper-dependent chain reaction (HDCR) [28] have been used for this purpose but face limitations, including low amplification efficiency (HDCR) and false positives (EXPAR) [29,30]. In contrast, specific-terminal-mediated polymerase chain reaction (STEM-PCR) is a promising approach which features a straightforward, cost-effective workflow compatible with widely accessible PCR platforms [31]. STEM-PCR has demonstrated sensitivity 20-fold higher than conventional BS conversion methods for site-specific DNA methylation detection [31], making it particularly suitable for applications in resource-limited settings, such as community hospitals. STEM-PCR can further be integrated with lateral flow detection (LFD) assays to create a visually interpretable and user-friendly platform [32,33]. Recent advancements in smartphone-based LFD readers have extended the utility of this technique from qualitative to semi-quantitative analysis, enhancing its accuracy and reliability [34].

In this study, we developed a highly specific and sensitive DNA methylation detection method by combining a methylation-dependent restriction endonuclease (GlaI) with STEM-PCR. Additionally, we introduced an integrated lateral flow device (LFD) featuring four detection channels to enable direct, on-site result reading. After STEM-PCR amplification, the LFD device facilitated efficient, cost-effective, and multiplexed detection. Using methylated *Septin 9*—an established biomarker for colorectal cancer (CRC)—as a case study, we evaluated the analytical and clinical performance of the assay. The method demonstrated a sensitivity of approximately 0.1% (10 copies of methylated template per reaction) and showed no cross-reactivity with 10,000 copies of unmethylated DNA. Furthermore, when applied to ten formalin-fixed paraffin-embedded (FFPE) tissue samples, the STEM-PCR-LFD assay exhibited 100% consistency with real-time STEM-PCR, confirming its robustness and reliability for clinical applications.

## 2. Materials and Methods

### 2.1. Reagents and Materials

Jurkat genomic DNA and methylated Jurkat genomic DNA were provided by Thermo Fisher Scientific, Waltham, MA, USA. A GlaI endonuclease was obtained from SibEnzyme Ltd., Novosibirsk, Russia, and Phanta Uc Super-Fidelity DNA polymerase was supplied by Sigma-Aldrich, Taufkirchen, Germany. Oligonucleotides were synthesized by Sangon Biological Corporation, Shanghai, China. Champagne Taq DNA polymerase and dNTPs were sourced from Vazyme Biotech, Nanjing, China. Lateral flow detection (LFD) strips for STEM-PCR amplicon detection were purchased from Ustar Biotechnologies Ltd., Hangzhou, China.

### 2.2. GlaI Digestion

The extracted DNA was treated with GlaI for methylation-specific digestion. The reaction mixture consisted of 10 mM Tris-HCl (pH 8.5 at 25 °C), 5 mM MgCl_2_, 10 mM NaCl, 1 mM 2-mercaptoethanol, and 8 units of GlaI in a total volume of 10 μL. The reaction was incubated at 30 °C for 45 min.

### 2.3. STEM-PCR Amplification

Real-time amplification via STEM-PCR was performed using a LightCycler 480 II (ROCHE, Indianapolis, IN, USA) in a 20 μL reaction volume. The mixture contained 10 nM tailored-designed foldable primer (TFP), 0.2 μM target-specific primer (TSP), 0.2 μM universal primer (UP), 0.1 μM Taqman probe, 0.3 mM dNTPs, 1 × PCR buffer, 1 U Champagne Taq DNA polymerase, 1 U UDG, and 10 μL of GlaI-treated DNA. The oligonucleotide sequences of primers and probe are shown in Table 1. The amplification cycle was as follows: initial denaturation at 95 °C for 5 min, followed by 10 cycles of 95 °C for 10 s, 66 °C for 90 s, and then 40 cycles of 95 °C for 10 s, and 65 °C for 30 s. Fluorescence was monitored during the annealing step, and the results were analyzed using Origin software (Version 2021, OriginLabs, Northampton, MA, USA).

### 2.4. Lateral Flow Detection (LFD)

The LFD strip consists of four main components: the sample pad, conjugate pad, nitrocellulose membrane, and absorbent pad. Prior to assembly, the sample pad was treated with a solution containing Tris-HCl (pH 8.0), 0.15 mM NaCl, and 0.3% Triton X-100. The absorbent pad was treated with 15 mM PBS (pH 7.5) containing 1% trehalose, 0.25% polyethylene glycol (PEG), and 0.1% bovine serum albumin (BSA), which was utilized to generate the capillary pressure necessary for liquid flow. The red, uncoated nanoparticles (Magsphere, Inc., San Francisco, CA, USA) were incubated for two hours at 20 °C in a glutaraldehyde buffer solution (pH 8.0) containing 10 mg/mL streptavidin. Subsequently, the streptavidin-coated nanoparticles were centrifuged at 10,000 rpm for ten minutes. The particles were then immobilized on a conjugate pad. On a nitrocellulose membrane (MDI Membrane Technologies, Inc., Adygeya, Russia), the anti-fluorescein isothiocyanate (anti-FTIC) antibody (Jackson ImmunoResearch Labs, West Grove, PA, USA) was applied to form the test line, while biotin was applied to form the control line. After pretreatment, the sample and conjugate pads were dried at 37 °C for 4–5 h and stored in a desiccator at room temperature. The various components of the LFD strip were assembled on a sticky back plate, with a 1.5–2 mm overlap to ensure efficient amplicon migration.

During detection, the amplified product was added to the sample pad, which was then immersed in 100 μL of 2× saline–sodium citrate (SCC) buffer. The amplicon was captured using streptavidin-conjugated red nanoparticles on the conjugate pad. The nanoparticles migrated by capillary action and accumulated at the test or control lines. A positive result was indicated by the appearance of both the test and control lines within five minutes.

For semi-quantitative analysis, images of the LFD results were captured using a mobile phone and processed with ImageJ software (Version 1.54). The LFD images were processed using ImageJ software (Version 1.54) with the following steps: First, the images were converted to 8-bit format, and the background was subtracted. Next, the measurements were configured to analyze the integrated density, and the image colors were inverted. Finally, the rectangle tool was used to measure the intensity of the test band. To minimize variations caused by photography, the LFD strips used for calibration and clinical studies were photographed in grouped batches, respectively.

## 3. Results

### 3.1. Principle of GIaI-Mediated STEM-PCR and Illustration of the Detection Process

**Figure 1:**
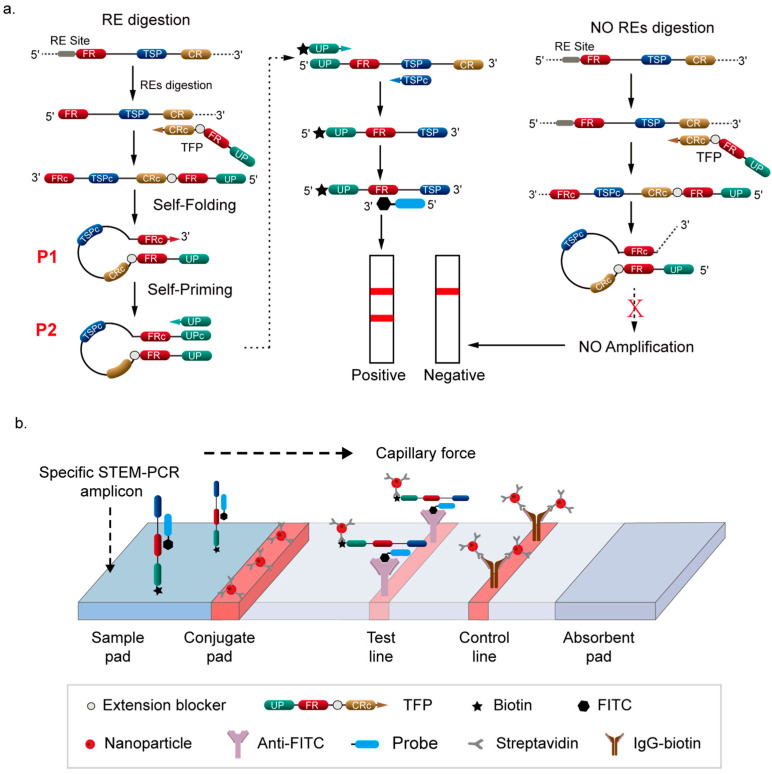
Overview of the STEM-PCR-LFD method for detecting DNA hypermethylation: (**a**) Only targets with the specific GlaI recognition site are digested by GlaI, generating fragments with a defined 5′ end that can self-prime into a hairpin structure, which is then amplified by STEM-PCR. (**b**) Working principle of lateral flow detection (LFD). Biotin/FITC dual-labeled amplicons are captured at the test line, producing a positive result.

Due to the strong selectivity of GlaI (5′-GmeCGmeC-3′/meCGmeCG), it specifically recognizes and digests only DNA templates containing methylation at the targeted sites. GlaI-mediated digestion results in the production of fragments from methylated DNA templates, while unmethylated DNA remains intact. For proof-of-principle analysis, a hypermethylated region of the *Septin 9* gene containing GlaI recognized sites was chosen for analysis based on previously study [31]. As illustrated in Figure 1a, when a DNA sequence with a GlaI restriction enzyme (RE) site is digested, it generates fragments with a specific 5′ end, which serve as templates to initiate the first stage of synthesis via a TFP. For the digested fragments, the synthesis process terminates at the 5′ end, producing a finite sequence that can self-fold (P1) and self-prime into a hairpin structure (P2). In contrast, for undigested or unmethylated fragments, the synthesis process continues, extending the 3′ end and preventing self-priming. Consequently, only the intact hairpin structure initiates the second stage of exponential amplification using the universal primer (UP).

For the LFD assay (Figure 1b), the UP used in standard STEM-PCR is labeled with biotin at the 5′ end, and the probe is labeled with FITC at the 3′ end. The probe can target the specific hybrid site on amplicons generated from exponential amplification mediated by UP. After STEM-PCR amplification, the biotin/FITC dual-labeled complex forms exclusively in reactions containing GlaI-treated methylated templates. The anti-FITC antibody captures the complex at the test line (T-line) of the LFD strip, providing a positive signal that indicates the presence of DNA methylation. If GlaI treatment or the methylated template is absent, no biotin/FITC dual-labeled complex is formed, resulting in a negative signal.

**Figure 2:**
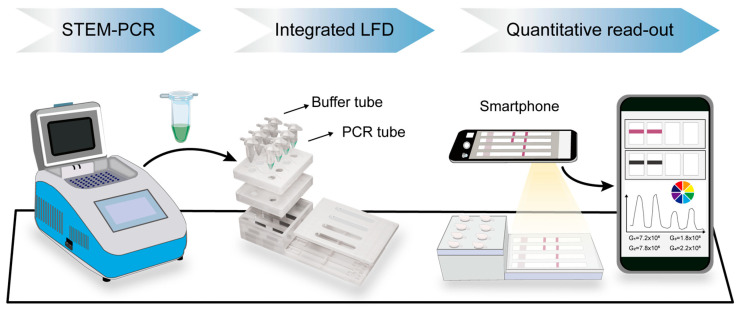
Work flow of STEM-PCR-LFD. Detection process begins from left to right: (1) STEM-PCR step on a bench-top thermal cycler. (2) Loading of PCR tubes containing amplicons and buffer tubes on integrated LFD device for results visualization. (3) Quantitative read-out of color intensity in test line photographed by smartphone, which is then analyzed with ImageJ software.

The entire detection process is illustrated in Figure 2. An integrated lateral flow detection (LFD) device, which includes four independent channels, is designed to facilitate simultaneous analysis of multiple samples. After performing STEM-PCR reactions on a bench-top PCR instrument, the PCR tubes containing the amplified DNA products are placed in a designated position in front of the buffer tubes on the LFD device. Then, both kinds of tubes were pressed down to corresponding position sequentially, where the pre-set pinpoints would pierce the bottom of tubes, allowing the running buffer to drive amplicons into microchannels for LFD.

In the presence of methylated DNA, the biotin/FITC dual-labeled amplicons are captured at the test line of the LFD strip. This interaction generates a visible positive signal, confirming the presence of DNA methylation. Conversely, the unmethylated template synthesizes structure with a 3′ end over-extends and prevents amplification by UP, which leads to the absence of biotin/FITC dual-labeled complex to show a negative result on LFD assay. This specificity of the assay ensures that only methylated DNA produces a signal on the LFD strip.

Additionally, a smartphone-based LFD reader is employed to extend the utility of the technique from a simple qualitative result to semi-quantitative analysis. This enhancement allows for more precise quantification of the methylation levels, improving the accuracy and reliability of the assay and facilitating its potential use in clinical diagnostics.

### 3.2. Optimization of STEM-PCR Assay

To construct an optimal STEM-PCR assay for DNA methylation detection with improved performance using LFD, several key experimental parameters were systematically explored, including varying concentrations of TFPs and the number of amplification cycles. The STEM-PCR method is designed to detect methylated DNA through a two-step amplification strategy. The first step involves the synthesis of an intermediate hairpin structure with a finite 3′ end, which is crucial for ensuring selective amplification of methylated templates. This hairpin structure is then amplified exponentially in the second step, allowing for highly sensitive detection of methylated DNA.

**Figure 3:**
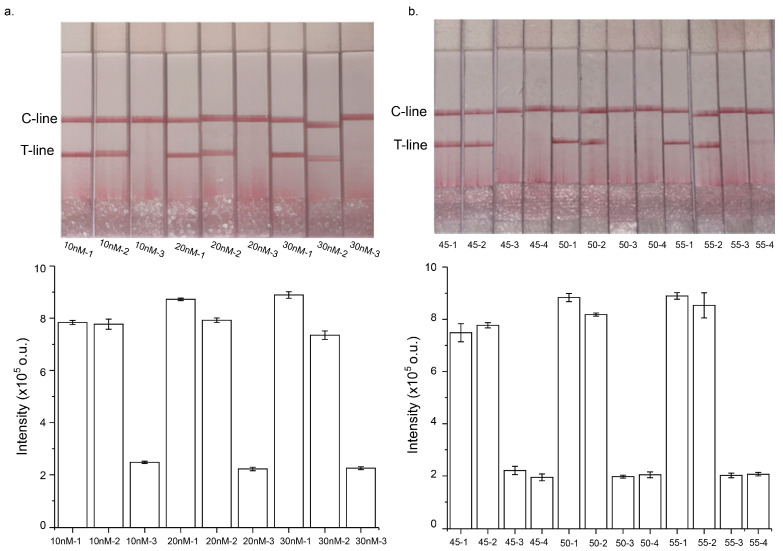
Optimization of the STEM-PCR-LFD assay: (**a**) Different concentration of TFPs from 10 nM to 30 nM. Under each concentration of TFPs, the detections were carried out using different GlaI-digested template: 1. 300 copies/reaction; 2. 30 copies/reaction; 3. ddH_2_O as negative control. (**b**) Different amplification cycle numbers of STEM-PCR process. Under each cycle number, different concentrations of GlaI-digested template were tested: 1. 300 copies/reaction; 2. 30 copies/reaction; 3. 10,000 copies unmethylated genomic DNA per reaction; 4. ddH_2_O as negative control. Representative photos (**top**) and the bar graphs for the intensity of test lines (**bottom**) for STEM-PCR-LFD. Error bar, 1 s.d. (n = 3).

In the first step, TFPs are used to hybridize with both digested and undigested DNA templates. To maximize the yield of the linear strand that self-folds into the hairpin structure, it is essential to optimize the concentration of TFPs. However, this must be performed without increasing nonspecific amplification, which could interfere with the results. To evaluate this, STEM-PCR assays were conducted with TFP concentrations ranging from 10 nM to 30 nM (Figure 3a). The results showed that changing the concentration of TFPs had little effect on the LFD results, suggesting that altering TFP concentrations did not influence the amplification efficacy of STEM-PCR. Moreover, the LFD results were consistent across various digested template inputs, confirming that TFP concentration did not significantly impact the outcome of the assay.

Next, the relationship between the number of amplification cycles and the LFD signal was investigated. The number of amplification cycles was varied from 45 to 55 to assess its effect on detection sensitivity. As shown in Figure 3b, LFD signals were able to clearly differentiate between methylated and unmethylated DNA templates across this range of cycle numbers. Interestingly, the signal generated from methylated templates exhibited minimal variance even as the amplification cycle number increased. This suggests that increasing the cycle number beyond a certain point did not substantially enhance the signal for methylated templates. These findings indicate that optimal results can be achieved without excessive amplification cycles, helping to streamline the detection process and reduce potential errors associated with over-amplification.

### 3.3. Performance of Septin 9 Methylation Analysis Using the STEM-PCR-LFD Assay

**Figure 4:**
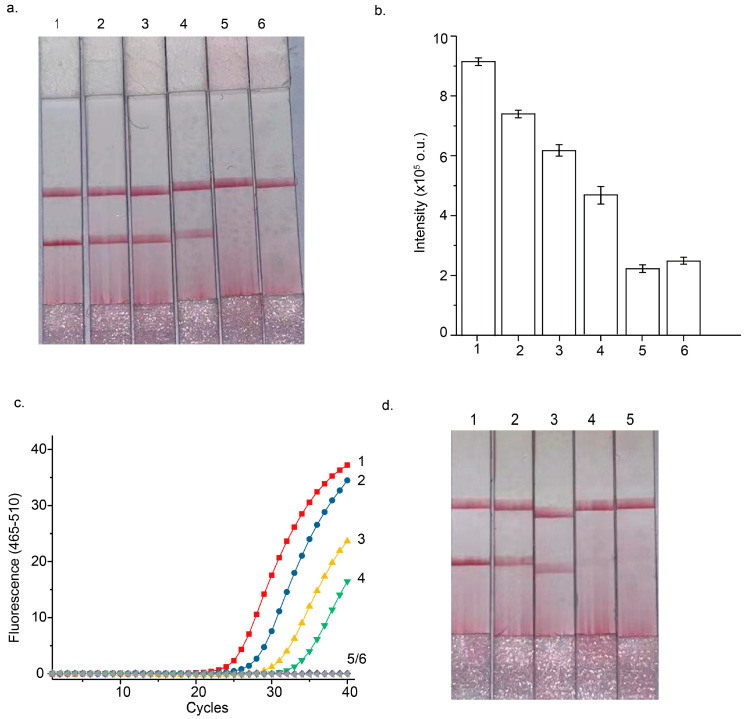
(**a**) Sensitivity of STEM-PCR LFD with serially diluted methylated templates: 1. 5000 copies/reaction; 2. 500 copies/reaction; 3. 50 copies/reaction; 4. 5 copies/reaction; 5. 10,000 copies unmethylated DNA per reaction; 6. ddH_2_O as negative control. (**b**) Bar graphs for the intensity of test lines in the (**a**). (**c**) Amplification plots of standard real-time STEM-PCR: 1. 5000 copies/reaction (red squares); 2. 500 copies/reaction (blue disks); 3. 50 copies/reaction (yellow triangles); 4. 5 copies/reaction (green inverted triangles); 5. 10,000 copies unmethylated DNA (turquoise lozenges); 6. ddH_2_O as a negative control (gray left triangles). (**d**) STEM-PCR-LFD with different GlaI-digested methylated templates ratios with 10,000 copies of unmethylated sequences as the background: 1. 10%; 2. 1%; 3. 0.1%; 4. 0%; 5. ddH_2_O as a negative control. Error bar, 1 s.d. (n = 3).

The analytical performance of the STEM-PCR-LFD assay for detecting *Septin 9* gene hypermethylation was systematically evaluated under optimized reaction conditions. To assess the sensitivity of the assay, serial dilutions of GlaI-pretreated methylated DNA were prepared, ranging from 5000 copies to as low as 5 copies per reaction. These dilutions were tested to determine the lowest detectable amount of methylated DNA. The results, shown in Figure 4a, revealed that the STEM-PCR-LFD assay could reliably detect methylated *Septin 9* down to 5 copies per reaction, demonstrating an exceptional level of sensitivity for DNA methylation detection.

To assess the specificity of the assay, unmethylated Jurkat genomic DNA was used as the wild-type background at a concentration of 10,000 copies per reaction. As shown in Figure 4a, the assay did not yield any positive signals when using the unmethylated DNA, even at high concentrations. This lack of cross-reactivity with unmethylated DNA confirms that the STEM-PCR-LFD assay is highly specific for detecting methylated DNA, with no false positives from unmethylated DNA. LFD signals displayed a declination when lowering the input of methylated DNA templates but still showed distinguishable differences from that of unmethylated background (Figure 4b).

Further validation of the sensitivity of the STEM-PCR-LFD assay was carried out by comparing it to the standard real-time STEM-PCR method under identical reaction conditions. The results presented in Figure 4c show that the STEM-PCR-LFD assay demonstrated sensitivity comparable to the real-time STEM-PCR, confirming that the LFD-based detection method is both reliable and efficient for methylation analysis.

To explore both sensitivity and specificity in greater depth, different percentages of GlaI-pretreated methylated DNA templates (ranging from 10%, 1%, 0.1%, to 0%) were prepared, with 10,000 copies of unmethylated DNA serving as the background in each case. The results, displayed in Figure 4d, demonstrated that the STEM-PCR-LFD assay could detect as low as 0.1% methylated DNA in the presence of a high background of unmethylated DNA, further highlighting the assay’s sensitivity. Importantly, even at such low methylation percentages, the assay did not show any cross-reactivity with unmethylated DNA, underscoring its high specificity. This ensures that the assay can effectively distinguish between methylated and unmethylated DNA.

### 3.4. STEM-PCR-LFD Assay of the Hypermethylated Septin 9 Gene Using CRC Samples

The proof-of-principle demonstration of the STEM-PCR-LFD assay was conducted using ten FFPE tissue samples from CRC patients, a common source of clinical samples for methylation studies. FFPE samples are often used in clinical settings because they are routinely archived for long-term storage and are rich in genetic material. The extracted genomic DNA from these tissue samples was first quantified using a Nanodrop spectrophotometer. For each assay, 10 ng of DNA was used as the template in both the standard real-time STEM-PCR and STEM-PCR-LFD assays.

**Figure 5:**
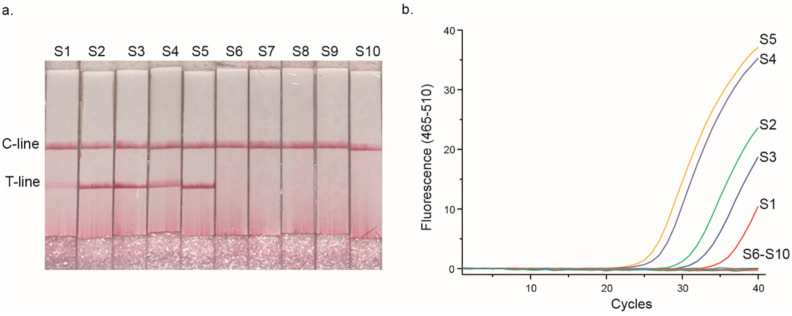
Results of ten clinical FFPE samples in the STEM-PCR assay: (**a**) STEM-PCR-LFD assay. (**b**) Standard real-time STEM-PCR. The samples were numbered as S1–S10.

The results of these assays revealed that both methods exhibited 100% consistency, with five of the ten samples showing positive signals that indicated the presence of methylated DNA (Figure 5). This result suggests that the STEM-PCR-LFD assay is capable of detecting DNA methylation with comparable accuracy to the well-established real-time STEM-PCR method. The high concordance between these two methods indicates that the STEM-PCR-LFD assay can be reliably used for the detection of DNA methylation in clinical samples. While the proof-of-principle results are promising, further clinical validation is essential to assess the performance of the STEM-PCR-LFD assay across a broader range of clinical contexts. Ultimately, this will pave the way for the clinical translation of the STEM-PCR-LFD assay, allowing it to be used as a reliable, cost-effective tool for early diagnosis and personalized treatment of cancer and other methylation-related diseases.

## 4. Discussion

Abnormal DNA methylation is a critical biomarker for various diseases, particularly cancer. While bisulfite sequencing (BS) remains the gold standard for methylation analysis, its limitations—including incomplete conversion and DNA degradation—pose challenges to accurate detection. Advanced DNA sequencing methods, such as Nanopore sequencing, next-generation sequencing (NGS), and single-molecule real-time (SMRT) sequencing, offer high precision but come with complexities, high costs, and data accuracy issues, further complicated by the need for expert bioinformatics analysis [35]. These challenges limit the widespread clinical applicability of these technologies.

In this context, we developed a bisulfite-free DNA methylation detection method—the GIaI mediated STEM-PCR-LFD assay—which combines the amplification efficiency of PCR with the high specificity of GlaI to detect methylated CpG sites. This method leverages LFD, allowing for rapid, cost-effective, and simple detection without the need for specialized equipment. By utilizing traditional thermal cyclers and integrating the STEM-PCR amplicons directly into the LFD device, we have minimized operational time and potential contamination risks. Moreover, the system allows for multiplexed detection, as four samples can be processed simultaneously.

Our results demonstrated that the STEM-PCR-LFD assay offers excellent limit-of-detection (down to 5 copies/reaction) and specificity, with no cross-reactivity observed from unmethylated DNA, even in the presence of a high background. We conducted ten tests at a target concentration of five copies per reaction, and nine of them yielded positive results. The assay’s performance was consistent with real-time STEM-PCR when applied to the methylation analysis of *Septin 9* in clinical samples. Importantly, the test’s simple workflow, rapid turnaround (within two hours), and ability to produce results visible to the naked eye or via a digital reader make it an ideal tool for clinical diagnostics.

The STEM-PCR-LFD assay represents a promising solution for DNA methylation analysis, particularly in settings where access to expensive sequencing equipment or skilled bioinformaticians is limited. This integration underscores the synergy between STEM-PCR and lateral flow tests, showcasing the smartphone as a versatile detector or enabling straightforward naked-eye interpretation for clear YES/NO outcomes. Given its accessibility, low cost, and reliable performance, this assay holds great potential for widespread clinical application, especially in community hospitals and resource-limited environments. Future studies with larger clinical cohorts will further validate its utility and expand its applications in disease detection and monitoring.

## Figures and Tables

**Table 1:** Primer and probe sequences for STEM-PCR for single-site methylation.

Name	Sequence (5′-3′)
*Septin 9*-TFP	TGTCAGCCAACGGTATTCATCTTTGCGCAGCTGGATGGG/iSp18/GTCCGCGGCCGCAGCA
*Septin 9*-TSP	Biotin-TGCCAGCCCAGCACCCA
*Septin 9*-probe	CCTTCGAAGTCCGAAATGA-FITC
*Septin 9*-UP	GCCTGTCAGCCAACGGTATTCATC

## Data Availability

Data will be made available on request.
